# Supplementary material for: GraphTS: Graph-represented time series for subsequence anomaly detection
Source: PLoS One. 2023 Aug 16;18(8):e0290092. doi: 10.1371/journal.pone.0290092 (PMC10431630; doi:10.1371/journal.pone.0290092)
Supplement: S1 Table — We show the selected parameters, the anomaly detection results of each method as well as its execution time for each dataset in UCR archive. Green cells/red cells designate dataset where methods detect/not detect the anomaly in that dataset. (PDF) [file pone.0290092.s001.pdf]

**Group**	**Dataset File**	**param.**	ℓ	**Running Time (s)**	
Name	#	w_g	[minL,maxL]	GraphTS	Series2Graph
ECG	109	70	[10,210]	19.77	476.58
	110	70	[10,210]	4.76	497.96
	111	70	[10,210]	7.74	497.83
	119	60	[10,100]	1.63	80.81
	120	60	[10,100]	1.56	50.02
	121	60	[10,166]	1.77	125.25
	122	60	[10,166]	2.98	215.58
	123	170	[10,450]	248.70	3016.23
	124	60	[10,166]	2.15	210.5
	125	60	[10,166]	2.80	215
	126	60	[10,166]	2.84	197.65
	163	20	[10,110]	3.22	288.97
	164	20	[10,110]	2.94	336.02
	165	20	[10,110]	3.48	332.60
	166	20	[10,110]	3.16	287.75
	178	100	[10,240]	19.12	504.02
	179	100	[10,240]	5.47	381.38
	180	100	[10,240]	8.02	433.05
	182	40	[10,175]	10.89	435.03
	183	40	[10,175]	10.57	378.55
	192	50	[10,105]	3.06	139.6
	193	50	[10,105]	1.77	135.81
	194	230	[10,280]	10.15	609.95
	195	80	[10,190]	13.55	487.35
	196	80	[10,190]	4.41	321.16
Internal Bleeding (IB)	132	25	[10,155]	1.72	42.39
	133	25	[10,155]	1.88	43.09
	134	25	[10,185]	3.80	55.69
	135	25	[10,185]	3.64	37.35
	136	25	[10,185]	1.62	34.13
	137	25	[10,185]	2.12	31.92
	138	25	[10,185]	2.49	27.01
	139	25	[10,185]	1.30	27.51
	140	25	[10,175]	3.25	36.39
	141	25	[10,175]	2.30	28.92
	142	25	[10,155]	2.62	41.47
	143	25	[10,165]	1.93	56.90
	144	25	[10,175]	2.04	22.18
Giat	127	150	[10,220]	2.08	39.56
	128	150	[10,220]	2.29	41.02
	129	150	[10,220]	2.15	40.05
	130	150	[10,220]	2.42	44.80
	131	150	[10,220]	1.53	40.76
	167	250	[10,315]	27.25	454.44
	168	250	[10,315]	33.38	427.33
	169	250	[10,315]	18.11	430.26
	170	250	[10,315]	56.24	423.35
	171	250	[10,315]	50.71	403.98
	172	250	[10,315]	53.79	458.91
	181	150	[10,345]	24.94	317.04

**Group**	**Dataset File**	**Param.**	ℓ	**Running Time (s)**	
Name	#	w_g	[minL,maxL]	GraphTS	Series2Graph
Insect	145	16	[10,100]	3.57	336.30
	146	16	[10,100]	2.53	332.74
	147	16	[10,100]	3.71	327.74
	148	16	[10,100]	2.36	316.64
	149	16	[10,100]	3.39	299.45
	150	16	[10,100]	2.26	311.80
	173	22	[10,100]	1.01	52.93
	174	22	[10,100]	1.03	47.14
	175	22	[10,100]	0.72	35.39
	176	22	[10,100]	0.71	66.08
	177	22	[10,100]	0.80	53.05
Respiration	184	20	[10,450]	103.64	6391.46
	185	20	[10,450]	173.36	5792.01
	186	20	[10,450]	133.99	4012.56
	187	20	[10,450]	227.36	7115.33
	188	20	[10,450]	245.49	5231.91
	189	20	[10,450]	220.79	5849.73
	190	20	[10,450]	144.67	5189.31
	191	20	[10,450]	244.67	6449.21
CHARIS	201	20	[10,100]	3.64	232.93
	202	20	[10,100]	2.37	198.30
	203	20	[10,100]	1.78	382.28
	204	20	[10,100]	2.56	187.83
	205	20	[10,100]	2.20	190.63
	206	20	[10,100]	1.28	102.81
	207	20	[10,100]	5.69	195.60
	208	20	[10,100]	3.22	366.36
Weather	113	15	[10,100]	0.68	36.28
	114	15	[10,100]	0.63	34.54
	115	15	[10,100]	0.65	33.52
	116	15	[10,100]	0.62	45.33
	117	15	[10,100]	0.71	43.59
	118	15	[10,100]	0.63	33.47
NASA	156	30	[10,100]	1.35	27.30
	157	30	[10,100]	1.22	27.24
	158	30	[10,100]	1.12	28.13
	159	30	[10,100]	1.31	27.54
	160	30	[10,100]	1.15	26.12
Other	112	30	[10,100]	1.66	31.99
	151	80	[10,280]	7.68	295.90
	152	96	[10,168]	2.34	182.78
	153	96	[10,168]	2.73	141.20
	154	96	[10,168]	1.73	128.15
	155	96	[10,168]	1.57	108.83
	161	20	[10,122]	1.20	63.36
	162	20	[10,122]	1.12	48.99
	197	130	[10,250]	11.72	316.72
	198	200	[10,250]	12.50	612.62
	199	200	[10,250]	9.83	770.02
	200	200	[10,250]	14.38	902.75
Total	100	-	-	2319.49	68294.86
