# Supplementary material for: GraphTS: Graph-represented time series for subsequence anomaly detection
Source: PLoS One. 2023 Aug 16;18(8):e0290092. doi: 10.1371/journal.pone.0290092 (PMC10431630; doi:10.1371/journal.pone.0290092)
Supplement: Attachment — Submitted filename: Response to Reviewers.docx [file pone.0290092.s002.docx]

Response to reviewers’ comments for:

GraphTS: Graph-Represented Time Series for Subsequence

Anomaly Detection

We thank the editor and all the reviewers for their constructive feedback to help improve this paper. Below please find the point-to-point responses to the comments raised by the reviewers. Accordingly, we highlighted our modifications in the new version.

**Response to Reviewer 1**

**Comment 1: the Related Work section is petite. Please add more work to this section and discuss it briefly.**

**Response:**

As suggested, we have added more related works (i.e., [39] [42] [43] [44]) and discussions to the Related Work section (see page 4, lines 121-157).

**Comment 2: Please provide a detailed description of your proposed model.**

**Response:**

We have added more overall description of the GraphTS method in the Introduction section of the revised version (see page 2, lines 46-60). Table 2 also summarizes the three steps of the GraphTS method, and we provide an example to explain the three steps in Figure 2. Accordingly, Sections 4.1 (Algorithm 1), 4.2 (Algorithm 2) and 4.3 (Algorithm 3) have been developed in details to explain the mechanism of each step.

**Comment 3: Specify the limitations and drawbacks of the proposed method.**

**Response:**

We have provided the limitation and future work for the proposed method (see page 17, lines 565-571)

**Comment 4: It is recommended to re-examine and design the Figures.**

**Response:**

We have re-examed all figures and replaced them with high-quality ones. Particularly, we have redesigned Figure 2 to ensure all subfigures are clear and readable.

**Comment 5: A deep and detailed comparison with other methods is mandatory.**

**Response:**

In the revised version, we have conducted a deep and detailed comparison with other methods (STOMP and Series2Graph) by adding more experimental results (see Figure 13 and S1 Table) on more datasets (see page 12, lines 412-419 and Table 4) and added the whole Section 5.5 for detailed explanation.

**Comment 6: The authors should also clarify the motivation and main contribution of applied approach more clearly in the introduction and conclusion sections.**

**Response:**

As suggested, we have revised the introduction and conclusion sections to clarify our motivation and contribution (see introduction section lines 86-103 on page 3 and conclusion section on page 17).

**Comment 7: The results and discussion section has to be improved, where more details of the achieved results should be stated clearly in this section. In addition, authors also have to provide some insight discussion of the results.**

**Response:**

We have improved the result and discussion section as suggested. We have added more detailed results and insight in the revised paper (see Section 5.5, Figure 13 and S1 Table)

**Response to Reviewer 2**

**Comment 1. Image quality is very poor. For example, it was hard to determine what's going on in (c1) and (c2) in Figure 2. The edge labels in (C3) of the same figure is almost illegible. Please replace the current images with high quality ones. Figure 3 is so blurry that the points and links between points are hard to distinguish. Better quality images would be help to understand the method well.**

**Response:**

We have re-examed Figure 3 and all of the other figures and replaced them with high-quality ones. Particularly, we have redesigned Figure 2 to ensure all subfigures are clear and readable.

**Comment 2. It's confusing that Z has been represented as a vector in Algorithm 1 (but it's actually a 2D matrix)? The symbols and notations used in the algorithm should be defined clearly.**

**Response:**

We have revised Algorithm 1 and provided a clear definition for matrix Z (see Algorithm 1 and lines 280-281 on page 8). We have also included a list of symbols in Table 1 on Page 7.

**Comment 3. The authors claim, in section 5.3, that the proposed method outperforms both STOMP and Series2Graph methods. This doesn't really give an accurate picture of the comparison. Out of 5 datasets, the proposed method outperforms Series2Graph on only one and performs the same on the remaining datasets. The authors should revise their claim and make it more specific. The one dataset on which the proposed method performs better, it does 20% better than Series2Graph which is satisfactory. However, if we consider the overall performance comparison shown in Figure 10, the proposed method does not do significantly better than Series2Graph. It would be more appealing if the authors used more datasets and could show that their proposed method performs better on at least about 50% of the datasets used in the experiment. However, considering the scalability factor, the proposed method does have some useful contributions.**

**Response:**

To enhance the evaluation, we have added more datasets (100 time series for seven groups of UCR datasets listed in Table 4) to compare our method with the Series2Graph method. Now the experimental results show that the proposed GrapTS outperforms Series2Graph in total accuracy while spending less runtime; that is, GrapTS absolutely excels Series2Graph in seven groups and achieves the same accuracy in two groups. (see Section 5.5, Figure 13 and S1 Table). We also have revised our claim in Section 5.3 (see lines 489-492 on page 15).

**Comment 4. Minor issues: Some grammar and spelling errors exist. For example, it should be "rest", not "reset", in the paragraph under Table 1.**

**Response:**

We have corrected the errors as suggested. Moreover, we checked the whole paper for grammar, spelling and punctuation mistakes and made the corrections in the revised version.
