# Supplementary material for: GraphTS: Graph-represented time series for subsequence anomaly detection
Source: PLoS One. 2023 Aug 16;18(8):e0290092. doi: 10.1371/journal.pone.0290092 (PMC10431630; doi:10.1371/journal.pone.0290092)
Supplement: Attachment — Submitted filename: Response to Reviewers comments.docx [file pone.0290092.s003.docx]

Response to reviewers’ comments for:

GraphTS: Graph-Represented Time Series for Subsequence

Anomaly Detection

We thank the editor and all the reviewers for their constructive feedback to help improve this paper. Below please find the point-to-point responses to the comments raised by the reviewers. Accordingly, we highlighted our modifications in the new version.

**Response to Reviewer 2**

**Comment 1: This article proposes a graph-based anomaly detection method for time series data. The proposed method, GraphTS, aims to do efficient detection of both recurrent and rare anomalies. I'll repeat from my first review that this article is well-organized, well-explained, and easy to read. I had several major concerns that the authors have addressed in their revised manuscript. One of which was about the lack of enough data and some claims they made that I didn't find convincing. However, I'm satisfied with their explanation and revision.**

**Response:**

Thank you for your positive feedback on our revised manuscript. We appreciate your contribution to improving our work.

**Response to Reviewer 3**

**Comment 1. Author should add keyword list. keywords list contain 5 to 8 words.**

**Response:**

As suggested, we have added a keywords list in the revised paper (see keywords section, page 1).

**Comment 2. Conclusion to be made more systematic and future scope to be elaborated more on technical features that are planned to be added in the proposed system in the near future.**

**Response:**

We have revised the conclusion section (see conclusion section, page 17) and elaborated on the future work in the revised paper (see lines 559-572 on page 17).

**Comment 3. author add more reference in introduction as below**

**1-Anomaly Detection via Correlation Clustering**

**2-Attack-Specific Feature Selection for Anomaly Detection in Software-Defined Networks**

**3-Two-phase classification: ANN and A-SVM classifiers on motor imagery BCI**

**Response:**

As suggested, we have added more related works (i.e., [45] [46] [47]) to the introduction section.

**Comment 4. Authors should further explain equations and maths. It is too hard to understand at the moment. Secondly, if these are general maths easily available on the internet, then authors should remove it and add reference instead.**

**Response:**

We have added explanation for Eq. (1) and cited reference for Definitions 1-4.

**Comment 5. The use of English language is fine, however, it is recommended to be checked once again.**

**Response:**

We have checked the whole paper for grammar, spelling and punctuation mistakes and made the corrections in the revised version.
